# Using Threshold Regression as an Approach to Incorporate Informative Missingness in Long Life Family Study Data

**DOI:** 10.1093/geroni/igab046.2520

**Published:** 2021-12-17

**Authors:** Ilya Zhbannikov, Konstantin Arbeev, Olivia Bagley, Yuriy Loika, Alexander Kulminski, Svetlana Ukraintseva, Eric Stallard, Anatoliy Yashin

**Affiliations:** Duke University, Durham, North Carolina, United States

## Abstract

Genetics of aging is important since aging is a major risk factor in most diseases. Variables describing physiological state and cognitive functioning that influence morbidity and mortality risks can serve as biomarkers of aging. They change with increasing age and the ways in which these variables change can also influence these risks. Missing data due to dropout or death create problems in longitudinal studies producing biased results especially if the gap between exams is relatively long, as is the case in the Long Life Family Study (LLFS). We applied the threshold regression model to LLFS data to investigate the vitality and its rate, which are conceptualized as latent variables characterizing health and longevity, and to cope with such a problem. We performed genome-wide association study by sex and age groups to discover genetic signals on these phenotypes. We found 11 variants from the DACT2 gene, p-values < 1E-6 and variants rs12151399 (p-value = 8.43E-8, intron variant, gene AGAP1, in females), rs27958 (p-value = 8.39E-8, intron variant, gene ARHGAP26, in males) showing associations with the vitality. Olfactory receptors showed significant enrichment among the group of males over 80 years for the rate of aging phenotype. Results showed that vitality and its rate differ among sex and age groups. This work is an important step toward understanding the processes of aging linking the vitality with individual genetics using data from deceased and living individuals.

